# Retinal hyperspectral imaging in the 5xFAD mouse model of Alzheimer’s disease

**DOI:** 10.1038/s41598-021-85554-2

**Published:** 2021-03-18

**Authors:** Jeremiah K. H. Lim, Qiao-Xin Li, Tim Ryan, Phillip Bedggood, Andrew Metha, Algis J. Vingrys, Bang V. Bui, Christine T. O. Nguyen

**Affiliations:** 1 Department of Optometry and Vision Sciences, University of Melbourne, Parkville, VIC 3010 Australia; 2 Florey Institute of Neuroscience and Mental Health, Parkville, VIC 3010 Australia; 3 SAXS/WAXS Beamline, ANSTO/Australian Synchrotron, Clayton, 3168 Australia; 4 Optometry and Vision Science, College of Nursing and Health Sciences, Flinders University, Bedford Park, SA 5042 Australia

**Keywords:** Retina, Diagnostic markers

## Abstract

Hyperspectral imaging of the retina has recently been posited as a potentially useful form of spectroscopy of amyloid-beta (Aβ) protein in the eyes of those with Alzheimer’s disease (AD). The concept of using the retina as a biomarker for AD is an attractive one, as current screening tools for AD are either expensive or inaccessible. Recent studies have investigated hyperspectral imaging in Aβ models however these studies have been in younger mice. Here we characterised hyperspectral reflectance profile in 6 to 17 months old 5xFAD mice and compare this to Aβ in isolated preparations. Hyperspectral imaging was conducted across two preparations of Aβ using a custom built bench ophthalmoscope. In the in vitro condition, 1 mg of purified human Aβ42 was solubilised and left to aggregate for 72 h. This soluble/insoluble Aβ mixture was then imaged by suspending the solution at a pipette tip and compared against phosphate buffered saline (PBS) control (n = 10 ROIs / group). In the in vivo condition, a 5xFAD transgenic mouse model was used and retinae were imaged at the age of 6 (n = 9), 12 (n = 9) and 17 months (n = 8) with age matched wildtype littermates as control (n = 12, n = 13, n = 15 respectively). In the vitro condition, hyperspectral imaging of the solution showed greater reflectance compared with vehicle (*p* < 0.01), with the greatest differences occurring in the short visible spectrum (< 500 nm). In the in vivo preparation, 5xFAD showed greater hyperspectral reflectance at all ages (6, 12, 17 months, *p* < 0.01). These differences were noted most in the short wavelengths at younger ages, with an additional peak appearing at longer wavelengths (~ 550 nm) with advancing age. This study shows that the presence of Aβ (soluble/insoluble mixture) can increase the hyperspectral reflectance profile in vitro as well as in vivo. Differences were evident in the short wavelength spectrum (< 500 nm) in vitro and were preserved when imaged through the ocular media in the in vivo conditions. With advancing age a second hump around ~ 550 nm became more apparent. Hyperspectral imaging of the retina does not require the use of contrast agents and is a potentially useful and non-invasive biomarker for AD.

## Introduction

Current biomarkers of Alzheimer’s disease (AD) are either invasive, expensive or have limited accessibility outside of research. This has led to searches for more accessible and lower-cost alternatives with recent advances in plasma biomarkers^1,2^ as well as the search for pathophysiological biomarkers in other locations such as the eye^3,4^. Although the presence of amyloid-beta peptide (Aβ) has previously been reported in the retinae of human post mortem eyes that were free of ocular disease, it was not confirmed if these donors indeed had AD^5^. Using curcumin as an amyloid binding agent for histology, retinal Aβ was detected in the post-mortem retinae of AD patients^6,7^. Co-labelling with Aβ-specific antibodies (11A5-B10, 12F4, 4G8, 6E10) and thioflavin-S support the presence of this hallmark in the retina^6,8^. However, other investigators using routine immunohistochemistry methods (primary antibody, 4G8 and 6F/3D) were unable to detect Aβ in the retinae of patients with confirmed AD^9–11^. Preclinical studies, in a range of mouse models genetically engineered to express human Aβ in the brain, find that Aβ also accumulates in the retinae^12–22^.

Given the vicissitudes in detecting Aβ in human retina, Koronyo et al.^6^ explored the possibility of in vivo fluorescence imaging of Aβ using curcumin as a contrast agent to bind insoluble and soluble fractions of Aβ, and were successful in demonstrating a scattered distribution of labelled deposits in living human eyes using scanning laser ophthalmoscopy. These investigators also used transmission electron microscopy to confirm that Aβ assembled into elongated fibrillar structures, reflecting depositions seen in the brain. Such results signal an encouraging step toward clinical utility; however, the need for a contrast agent reduces the practicality of retinal imaging as a screening tool, particularly with regards to cost, time and potential side-effects.

To date, there have been several attempts to exploit the fibril-associated molecular deposition of Aβ to permit detection without the use of extraneous contrast. In post-mortem eyes, Campbell and co-workers distinguished AD retinae from controls in a canine model of AD using non-invasive imaging of retinal birefringence arising from fibrillar Aβ deposition with the aid of cross polarisers^23–25^.

The scattering of light by accumulations of small, structured molecules in biological tissues can also manifest useful wavelength-dependencies^26,27^. In this regard, hyperspectral imaging (HSI) employs the principles akin to standard spectroscopy, which is used to determine the constituents of a sample or material by analysing absorption of specific narrow band wavelengths. Initially developed in the late 1960′s for remote sensing; HSI has gained traction in other fields ranging from biomedical imaging to archaeology. More and Vince^27^ suggested a scheme to exploit HSI for amyloid detection in AD. Using a hyperspectral darkfield microscope with a broadband light source to image an ex vivo preparation of SH-SY5Y human neuroblastic cells, they showed that the addition of soluble Aβ42 (250 nM) produced a reduction in the spectra for wavelengths between 450 and 530 nm. Applying this technique to ex vivo brain slices from human AD and ex vivo retinae from young APP/PS1 mice (without brain plaques), the authors showed a reduction in spectral reflectance at shorter wavelengths (< 580 nm) compared with control tissue^27^. These changes were attributed to the presence of soluble amyloid, small particles which are known to scatter light in tissue in a wavelength dependent fashion^26^.

In a follow-up to their ex vivo study, the authors modified and applied their approach for in vivo imaging of APP/PS1 transgenic AD mouse retinae^28^. Using a “topical endoscopic fundal imaging (TEFI) system” they identified decreased reflectance at short wavelengths (< 570 nm)^28^ in 7 month old APP/PS1 mice. They attributed these reflectance changes to increased soluble Aβ, as insoluble Aβ deposits are only sparsely found in retina and in older (9–11 months) APP/PS1 mice^7,29^.

More recently, Hadoux et al.^30^ compared hyperspectral signatures in 15 people with mild cognitive impairment (MCI) and high Aβ burden quantified using positron emission tomography (PET) with 20 age-matched controls. In this cross-sectional study, using modelling to account for individual variation in factors such as macular pigmentation and retinal oxygenation, they were able to detect a difference in the spectral profile between the MCI and control group. Hadoux et al.^30^ also found a similar difference in retinal reflectance between 9 to 14 month old controls and 5xFAD mice, a model of AD^31^.

The data of More et al.^28^ show that APP/PS1 mice retinae indicate progressively more scatter with age (3 to 8-month-old), which they suggest is consistent with increasing soluble amyloid content. Mice used in More et al.^28^ were relatively young, and as such whether similar differences are still detectable at much older ages is not known. How advancing age-related factors such as optical media and retinal changes impact hyperspectral reflectance profile has yet to examined. By examining 5xFAD mice, we hope to show that HSI reflectance profiles provide useful information in a widely used murine model of amyloid deposition. Here, we examine age-related change to the HSI reflectance profile in 6, 12 and 17 months of age wildtype and 5xFAD mice which approximate preclinical, moderate and severe AD stages^32–34^ and exhibit retinal Aβ staining^22^.

## Methods

Two preparations of Aβ underwent hyperspectral imaging on the same platform. Firstly, an in vitro preparation of purified human Aβ42, and secondly an in vivo preparation in a transgenic 5xFAD mouse model. Both preparations and all ages^22^ contained a mixture of soluble and insoluble Aβ. All experimental procedures abided by the “Australian code of practice for the care and use of animals for scientific purposes” as set out by the National Health and Medical Research Council of Australia (2013). Ethics approval was obtained from the Howard Florey Institute Animal Ethics Committee (Approval number: 13-068-UM). In addition, permission was sought from the Office for Research Ethics and Integrity for a notifiable low risk dealing approval (IBC reference: 2013/050) for the use of genetically modified organisms in accordance with the Australian Gene Technology Act (2000) and Gene Technology Regulations (2001). This study was carried out in compliance with the ARRIVE guidelines^35^. Experimenters were blinded to the treatment groups during data collection and analysis. 5xFAD and wild type littermates were randomised within blocks for time and day of experimentation.

### In vitro preparation of Aβ42

Powdered purified human Aβ42 (Sigma A9810, St Louis, MO, USA) weighing 1 mg was suspended in 100 μl of 1 M of sodium hydroxide (NaOH) and incubated for 3 min at room temperature. The solution was diluted in 350 μl of dH_2_O and sonicated in a water bath at room temperature for 5 min. For neutralisation, 50 μl of 10 × phosphate buffered saline (PBS, pH 7) was added. The solution was then centrifuged (Centrifuge 5415D, Eppendorf AG, Hamburg, HH, Germany) at 14,000 × *g* for 10 min. The supernatant (450 μl) was removed to allow for the exclusion of undissolved particulate matter. The optical density of the supernatant was measured at 214 nm using a spectrophotometer (UV–VIS 2700, Shimadzu, Chiyoda-ku, Tokyo, Japan). This was used to determine the concentration using the Beer-Lambert law (Eq. 1).

1 $$A= \varepsilon . c. l$$

where A = absorbance, ε = extinction coefficient (M^-1^.cm^-1^), *c* = concentration (M) and *l* = path length (cm). Using an extinction coefficient for Aβ1-42 of 95,452 M/cm^36^ and a known path length of 1 cm, we were able to determine the concentration of the solution, which was typically 70–80% of the peptide.

Using a combination of amyloid-binding dye (Thioflavin-T), size exclusion chromatography and transmission electron microscopy, Ryan and colleagues showed that soluble Aβ prepared this way aggregated after 20-h^37,38^. To ensure that the solution contained a mixture of aggregated components, the Aβ solution was allowed to rest at 4 °C for a minimum of 72 h prior to measurement. This produced a solution that contained both soluble and insoluble Aβ.

### In vivo Aβ assessment in 5xFAD mice

The Tg(APPSwFlLon,PSEN1*M146L*L286V)6799Vas/Mmjax) mouse model of AD, also known as “5xFAD” was used in this study due to its well characterised phenotype^31^. These mice were bred on a congenic C57BL/6 J background and do not contain the Pde6brd1 retinal degeneration allele. This model has 5 familial human AD genes and express human APP and Aβ at an early age, allowing for the full-time course of AD development to be studied in a relatively short timeframe. A subset of the animals assessed in this study have been shown to exhibit soluble and insoluble Aβ in the retina and brain^22^ which is in agreement with the literature^14,30,39^. Retinal hyperspectral imaging was conducted at 3 ages, namely 6 (5xFAD, n = 9; WT n = 12), 12 (5xFAD, n = 9; WT n = 13) and 17 months of age (5xFAD, n = 8; WT n = 15). These timepoints were chosen as behavioural studies in 5xFAD mice have indicated that they approximate preclinical, moderate and severe AD, respectively^32–34^. The sample sizes are in agreement with previous power calculations and hyperspectral analysis from 5xFAD mice which spanned the middle age range (9 to 14 months old) of this group^30^. Mice were group housed in well-ventilated conditions where the ambient temperature was maintained at 20 °C. A 12 h light/dark cycle was used. Food (Baraston mouse pellets, Ridley Corporation, Melbourne, VIC, Australia) and water were provided ad libitum. Ambient light levels were maintained at a low average level of 50 lx to prevent retinal damage^40^ and minimise stress^41^.

*Animal handling and anaesthesia:* Prior to procedures mice were anaesthetised using ketamine : xylazine anaesthesia (60 : 5 mg/kg, i.p. Therapon, Burwood, VIC, Australia). Topical anaesthetic and mydriatic eyedrops (Alcaine 0.5%, Mydriacyl 0.5% respectively, Alcon Laboratories, Frenchs Forest, NSW, Australia) were instilled. Animals were then placed onto a heated platform to maintain body temperature. All imaging was completed within 20 min of anaesthesia in order to maximise anterior eye clarity. During imaging, eyes were kept moist using Genteal gel (Alcon Laboratories, Frenchs Forest, NSW, Australia) and a coverslip, which prevents the development of cataracts and corneal dehydration.

### Hyperspectral imaging platform

The custom-built bench ophthalmoscope uses a 150 W xenon light source with a fast switching monochromator (Polychrome V, Till Photonics, Hillsboro, OR, USA) as a hyperspectral light source (Supplementary Fig. 1). The light source has an intensity profile which varied by wavelength (Supplementary Fig. 2). The light source was placed perpendicular to the front of the eye or the in vitro preparation thereby providing bright-field flood illumination of the specimen imaged. A semi-reflective pellicle beam splitter (BP245B1, ThorLabs, Newton, NJ, USA) directs 45% of the incident light into the rodent eye. Light from the retina is returned through diffuse reflection and passes through the pellicle again. A condensing lens focuses the image directly onto a scientific complementary metal oxide semiconductor (CMOS) chip (Andor Technology, Belfast, UK). The light source and monochromator is capable of supplying visible light for retinal imaging using 10 nm bandwidths and is able to sweep out 1 nm steps centred from 320 to 680 nm. Settling time for each step was 100 ms, so that a full spectral series of 16-bit monochromatic images at each of 361 wavebands were acquired in 36.1 s.

**Figure 1:**
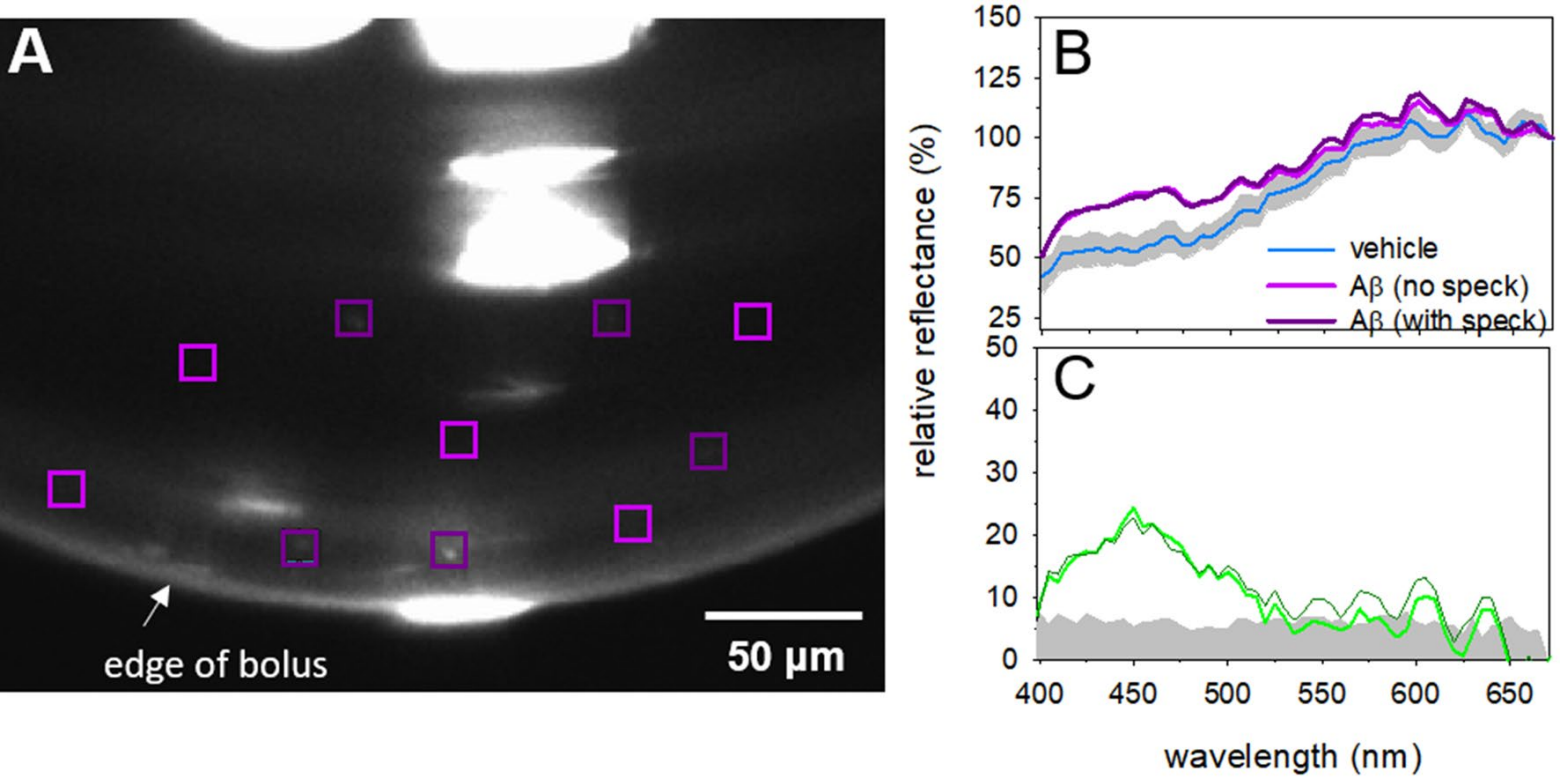
Spectral profile of isolated human Aβ42. **a** A representative image of a 10 µl droplet of 250 μM Aβ solution. Regions of interest (10 × 10 pixels, coloured squares) were chosen to either include visible Aβ aggregations (white specks, dark purple) or avoid visible Aβ aggregations (light purple). **b** Hyperspectral profiles of in vitro Aβ preparation either including visible aggregations (dark purple, n = 10 ROIs) or excluding visible aggregations (light purple, n = 10 ROIs) and PBS vehicle control (blue line with grey shaded region representing, mean ± 95% CL, n = 10 ROIs). **c** The difference between the Aβ preparation and control are represented in the average residual plot (dark green includes visible aggregates, light green excludes visible aggregates) with the grey area representing 95% CI around the vehicle group only to aid visualisation.

**Figure 2:**
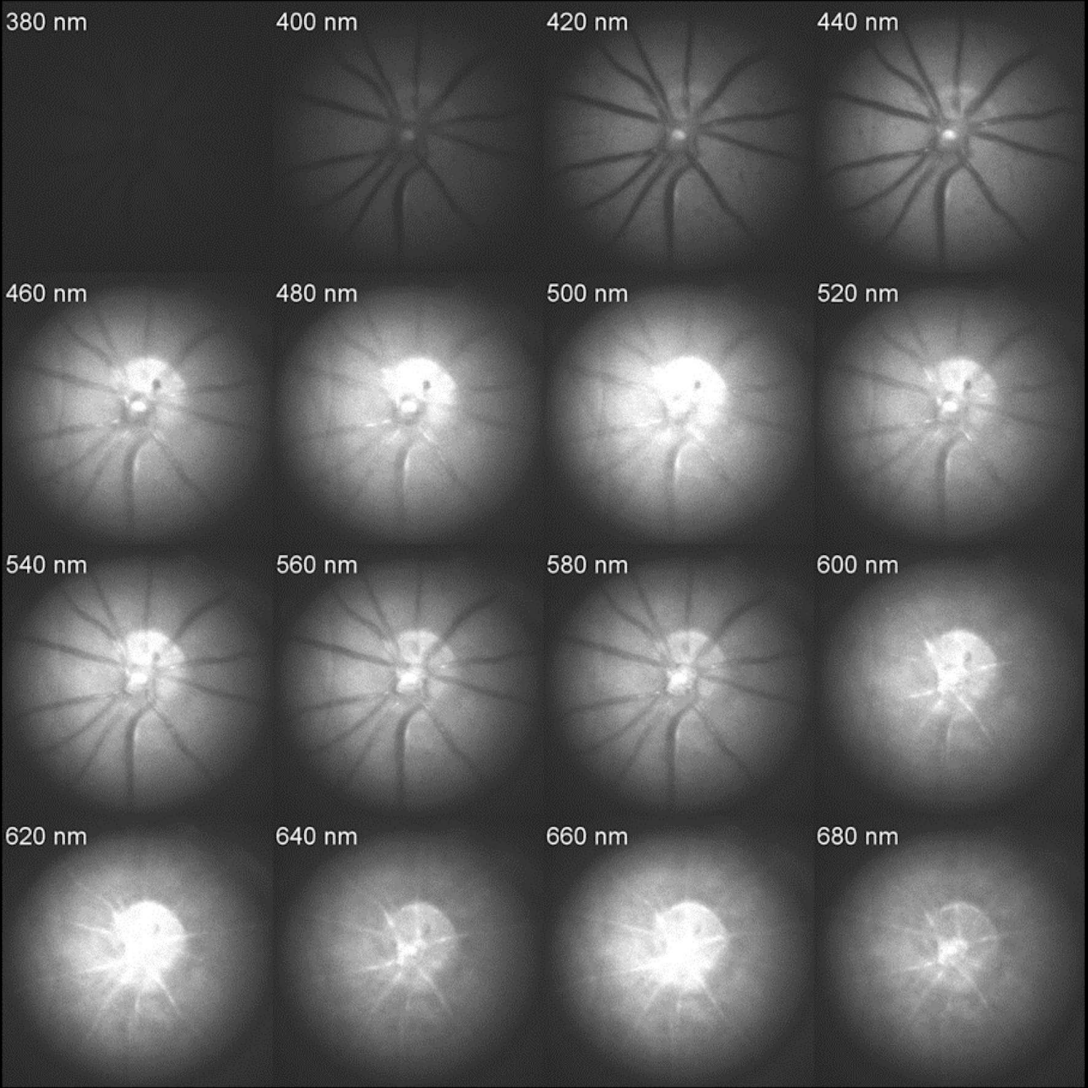
Retinal wavelength series in a representative 6-month-old WT mouse. Monochromatic images were obtained by illuminating the retina using wavelengths 380–680 nm (only 20 nm intervals pictured). Notable wavelengths include 480 nm, where the overall image is brightest; 560 nm, where arteries and veins are equally dark; 600–620 nm, where light absorption by oxygenated haemoglobin cause arteries to appear brighter than veins; and 680 nm, where the retinal regions between major blood vessels appear evenly illuminated due to reduced absorption by melanin.

Hyperspectral images collected by the bench ophthalmoscope are influenced by the light output from the Polychrome V monochromator as well as the pellicle reflectance (light input arm) and transmittance (imaging arm). To correct for changes accrued in the imaging arm we divided by the pellicle transmittance profile as specified by the manufacturer; to correct for changes accrued in light input we used a power meter to measure irradiance in the plane of the pupil (therefore accounting for both the pellicle’s reflectance to the eye and spectral variations in the polychrome light source). All raw in vivo and in vitro spectra were divided by these factors (Eq. 2). See Supplementary Fig. 2 for further details.

2 $$Corrected intensity \left(\lambda \right)= \frac{Raw intensity (\lambda )}{Pellice transmittance \left(\lambda \right)*Light input (\lambda )}$$

To establish the safety of the device on the retina, a digital photometer (Model IL1700, International Light Technologies Inc., Newburyport, MA, USA) was used to compute the power density at the brightest wavelength (475 nm), which produced an output of 708.8 μW/cm^2^. The maximum pupil diameter measured was 2.3 mm, which approximates 30 μW of light reaching the retina. This level is considered safe based on the international electrotechnical commission 60,825 values for maximum permissible exposure^42^.

### Image processing

Images were analysed using open source image analysis software, FIJI^43^. In vitro and in vivo images were exported as 16-bit greyscale Tag Image File Format (TIFF) stacks comprised of 361 images. Stacks were registered (using StackReg: Translation) to ensure that the same region of interest (ROI) was analysed in each image.

For in vitro Aβ samples, square (10 × 10 pixels) ROIs including and excluding visible amyloid aggregates were analysed for spectral intensity (Fig. 1a). For in vivo analysis, whole retinal images were manually masked to remove the major blood vessels and the optic nerve. The remaining image which represents retinal tissue and small blood vessels was analysed for hyperspectral reflectance. For quantification of in vitro and in vivo samples, hyperspectral profiles were first baseline corrected by subtracting the baseline light intensity at 380 nm (*λ*_*base*_), and then normalised to the reference wavelength (*λ*_*ref* =_ 670 nm). 670 nm was chosen as our in vitro analysis showed that Aβ had a similar light intensity to vehicle at this wavelength (Fig. 1). This wavelength has also previously been shown to be less sensitive to scatter in biological tissues^28^, though this may also be the case for longer wavelengths^27^.

To topographically visualise spatial distribution of hyperspectral change across the retina in 5xFAD mice, masked images were baseline corrected by subtracting the baseline image (*λ*_*base* =_ 380 nm) from the image stack. Processed images were analysed for Aβ (*λ*_*Aβ*_ = 475 nm as determined from the in vitro and in vivo Aβ imaging, Fig. 1b, 4a respectively) and expressed relative to the reference wavelength (*λ*_*ref*_ at 670 nm; Eq. 3).

3 $$\mathrm{Spectral \Delta } = \frac{({\lambda }_{A\beta }-{\lambda }_{base})}{({\lambda }_{ref}-{\lambda }_{base})}$$

**Figure 3:**
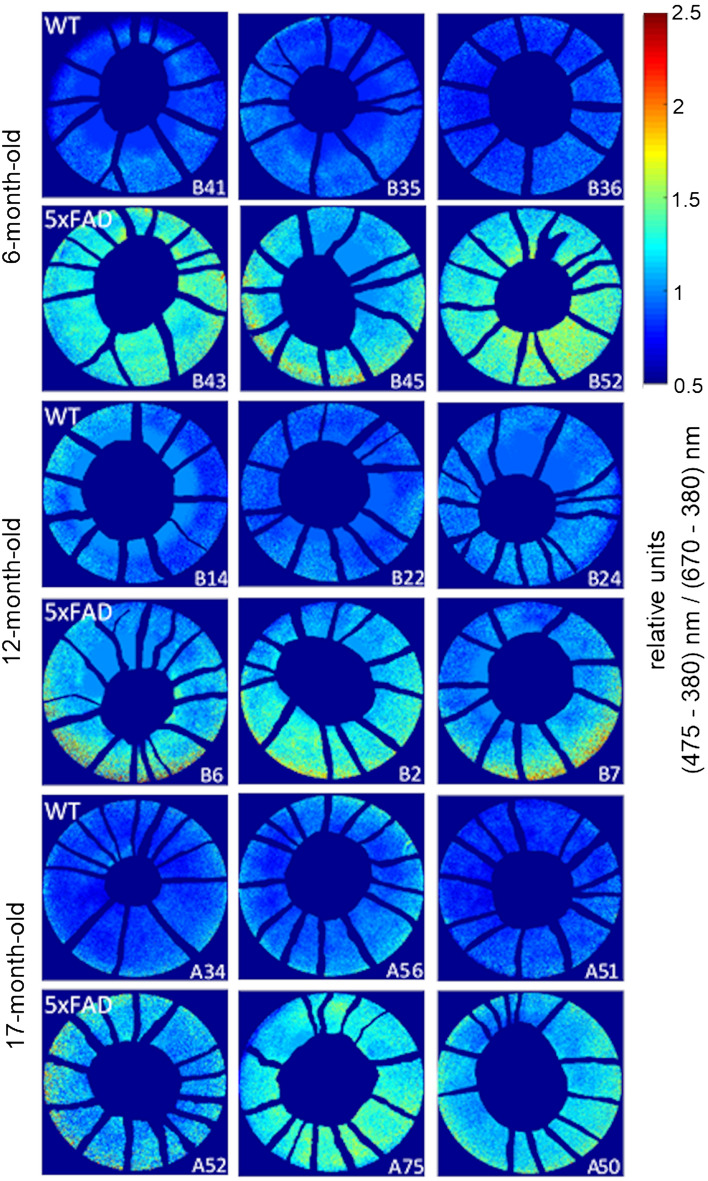
*En face* retinal images colour coded to reflect areas of greatest spectral difference at 475 nm. Difference plots were generated by expressing the image acquired at 475 nm relative to the one acquired at 670 nm. Wild type littermates in general showed a ratio closer to 1 (cooler colours), whereas 5xFAD retinae showed ratios of around 1.5 (warmer colours). This difference allows 5xFAD retinae to be visually discernible from WT retinae.

The resulting ratio plots (Fig. 3) are colour coded to produce a topographical heat map (MATLAB R2013a, MathWorks, Natick, MA, USA) in order to highlight regions containing maximal differences in the spectra between 5xFAD and WT.

## Statistical analysis

Normality of the data was established using a Kolmogorov–Smirnov test. A Grubbs test was used to identify outliers. Statistical comparisons (Supplementary Table 1 & 2) were carried out using repeated measures two-way ANOVA to establish differences between combinations of Aβ model and wavelength. Geisser-Greenhouse correction was applied as sphericity was not assumed. Post-hoc analysis was corrected for multiple comparisons by controlling the false discovery rate using a two stage linear step up procedure appropriate for overall recommendations based on multiple inferences as per Benjamini et al.^44,45^ An alpha of 0.05 was considered to be statistically significant. Statistical analyses were carried out in Prism 6 (GraphPad, San Diego, CA, USA).

## Results

### Spectral profile of in vitro Aβ42

The first aim was to determine if isolated human Aβ42, containing a mixture of soluble and insoluble components, had a HSI profile (380–680 nm) distinguishable from vehicle. Two 10 μl boli of Aβ42 (2 mg/ml) and PBS vehicle were suspended from the tip of a pipette. Figure 1a illustrates that visible aggregates (dark purple ROIs) can be seen in the Aβ solution. These are approximately ~ 1–2 µm in size which would represent a large aggregate. The surrounding darker areas may contain smaller insoluble and soluble aggregates below the magnification resolution of the system. As such to ensure both soluble and insoluble Aβ was captured, 5 ROIs (10 × 10 pixel size) were selected that contained a visible Aβ aggregate (insoluble Aβ with the surrounding background region including soluble Aβ) and 5 ROIs selected that did not contain a visible Aβ aggregate (soluble predominant, no visible insoluble specks seen) were selected from each Aβ drop. Note that it is likely that both types of ROIs (“with speck” and “no speck”) contain a mixture of soluble and insoluble Aβ, but the “with speck” ROIs include a higher proportion of insoluble given the larger visible aggregate. Phosphate buffered saline (PBS) was measured as vehicle control, with 5 ROIs in each of two droplets assessed.

The HSI reflectance for Aβ were similar between those regions of interest including visible aggregates (with speck) and excluding visible aggregates (no speck) (Fig. 1b, Supplementary Table 1, interaction p < 0.001). Moreover, both Aβ regions of interest showed significantly greater HSI reflectance at than vehicle at shorter wavelengths. The largest and most significant difference between Aβ ROIs and vehicle ROIs occurred between 435 to 480 nm (Fig. 1c, Supplementary Table 1, p = 0.0021 to p = 0.0054). Spectral reflectance at wavelengths from 415 to 505 nm were significantly different from control (post hoc, p < 0.05, Supplementary Table 1).

### Spectral profile in an in vivo model, 5xFAD mice

Unlike the in vitro preparation, we were unable to visualise discrete aggregates or plaques in the 5xFAD mouse retinae using HSI (Figs. 2, 3). Other studies using other Aβ animal models have employed contrast agents to identify amyloid plaques^7,46^. Whether the lack of discrete plaque visualisation was due to the distribution or concentration of Ab oligomers and plaques in these mice^22^ or the imaging approach employed is unclear. Nevertheless, to achieve an overall gauge of whether the retinal hyperspectral profile was altered in 5xFAD mice, we analysed whole retinal images to generate spatial maps of relative reflectance differences at short wavelengths (Eq. 3).

As seen from the wavelength series (Fig. 2), the optic nerve and the larger blood vessels in the retina exhibit distinct hyperspectral signatures. There is a wealth of literature showing that the spectral characteristics of the large blood vessels^47,48^ and optic nerve are affected by changes in blood volume, oxygen saturation^49,50^ connective tissues. Given that Aβ is thought to deposit in the neural retina (although some is found in vessel walls)^6^, the major blood vessels and the optic nerve area were masked prior to further analysis (Fig. 3).

**Figure 4:**
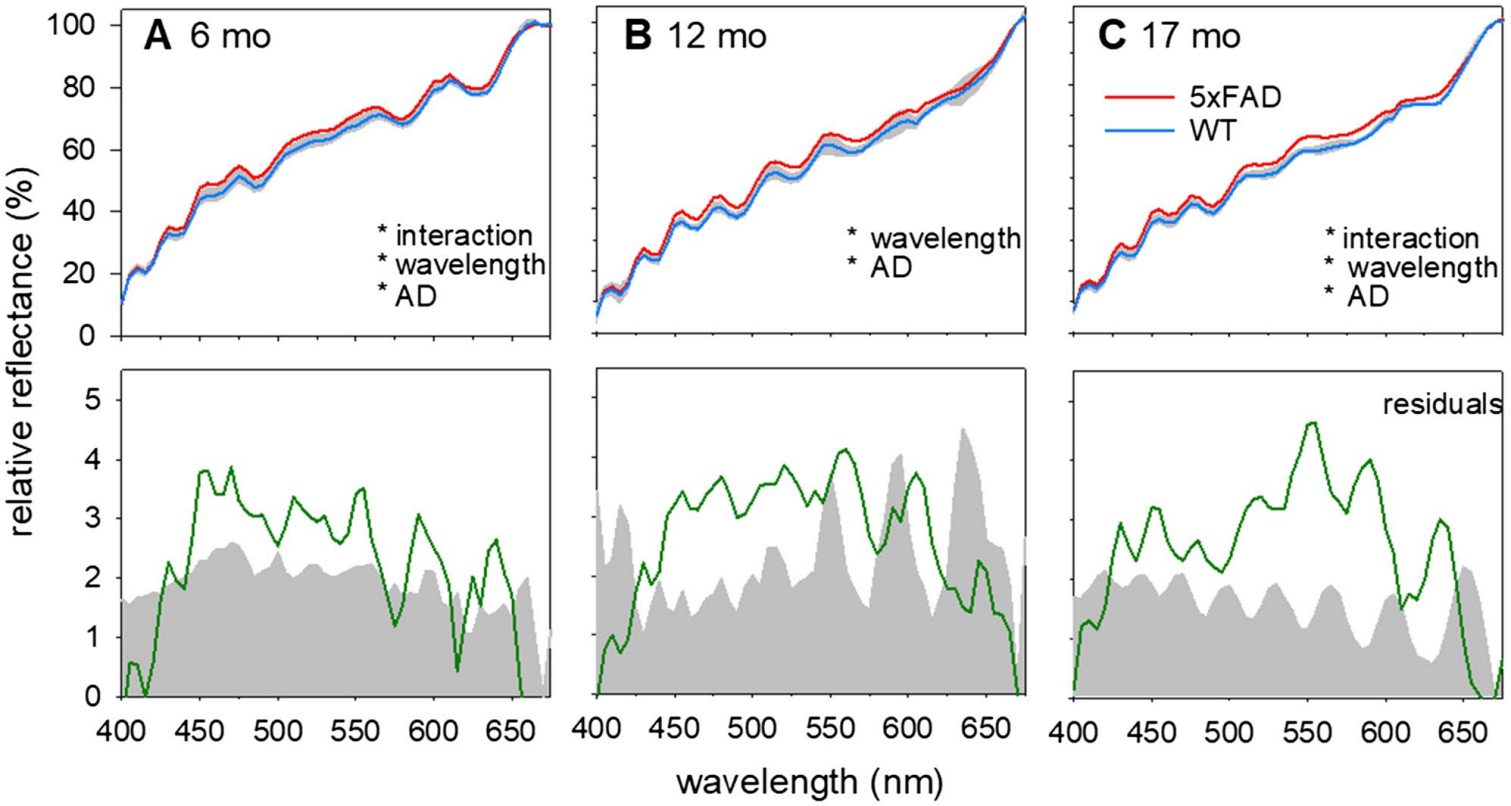
Hyperspectral profile of 5xFAD shows increased relative reflectance compared to WT. In vivo imaging of 5xFAD and WT retinae using hyperspectral imaging (n = 8–15/group) shows a separation between 5xFAD (red trace) and WT (blue trace) at 6 (**a**), 12 (**b**) and 17 (**c**) months of age. This difference is plotted as residuals (green line). *Significant difference using two-way ANOVA; Grey shaded regions, 95% confidence limits of WT only to aid visualisation.

After the mask was applied, reflectance at 475 nm was expressed as a ratio of that at 670 nm for each image pixel (Fig. 3). 475 nm was chosen as the criterion wavelength as it was representative of increased reflectance when referenced to 670 nm in both in vitro (Fig. 1) and in vivo preparations (Fig. 4 shows the full profile). This wavelength was similar to More et al.^28^ who used a criterion of 480 nm, with 670 nm as the reference wavelength. Figure 3 shows that across all ages 5xFAD mice generally exhibit higher relative reflectance at 475 nm (warmer colours) compared to WT mice. The increased reflectance did not appear to favour specific retinal locations. As our imaging system has a field of view limited to 20 degrees, we were unable to determine if there were greater spectral differences in the peripheral retina compared with the central retina.

Given the lack of region-specific difference, the relative reflectance of the whole retinal image (without blood vessels and the optic nerve) was averaged. Figure 4 compares the hyperspectral profiles between 5xFAD and WT at the three ages. The difference between 5xFAD and WT are plotted as residuals in the lower panels. At all 3 ages, there was increased reflectance in 5xFAD compared with WT across the visible spectrum. At 6 months of age, there was a significant interaction effect between wavelength and genotype (Supplementary Table 2A, p < 0.0001) with a peak difference found at 470 nm and 5xFAD mice deviated from WT fairly consistently between 450-490 nm (Fig. 4A, Supplementary Table 2A). At 12 months, increased reflectance toward shorter wavelengths (< 490 nm) and a second hump around ~ 550 nm was also observed (Fig. 4b, Supplementary Table 2). Using two-way ANOVA, the spectral differences were explained by wavelength and genotype (wavelength effect, p < 0.0001, genotype effect, p < 0.05). At 17 months of age, the greatest differences were observed at longer wavelengths ~ 555 nm spanning a 510-560 nm band and a smaller peak at shorter wavelengths (Fig. 4c, Supplementary Table 2C).

**Figure 5:**
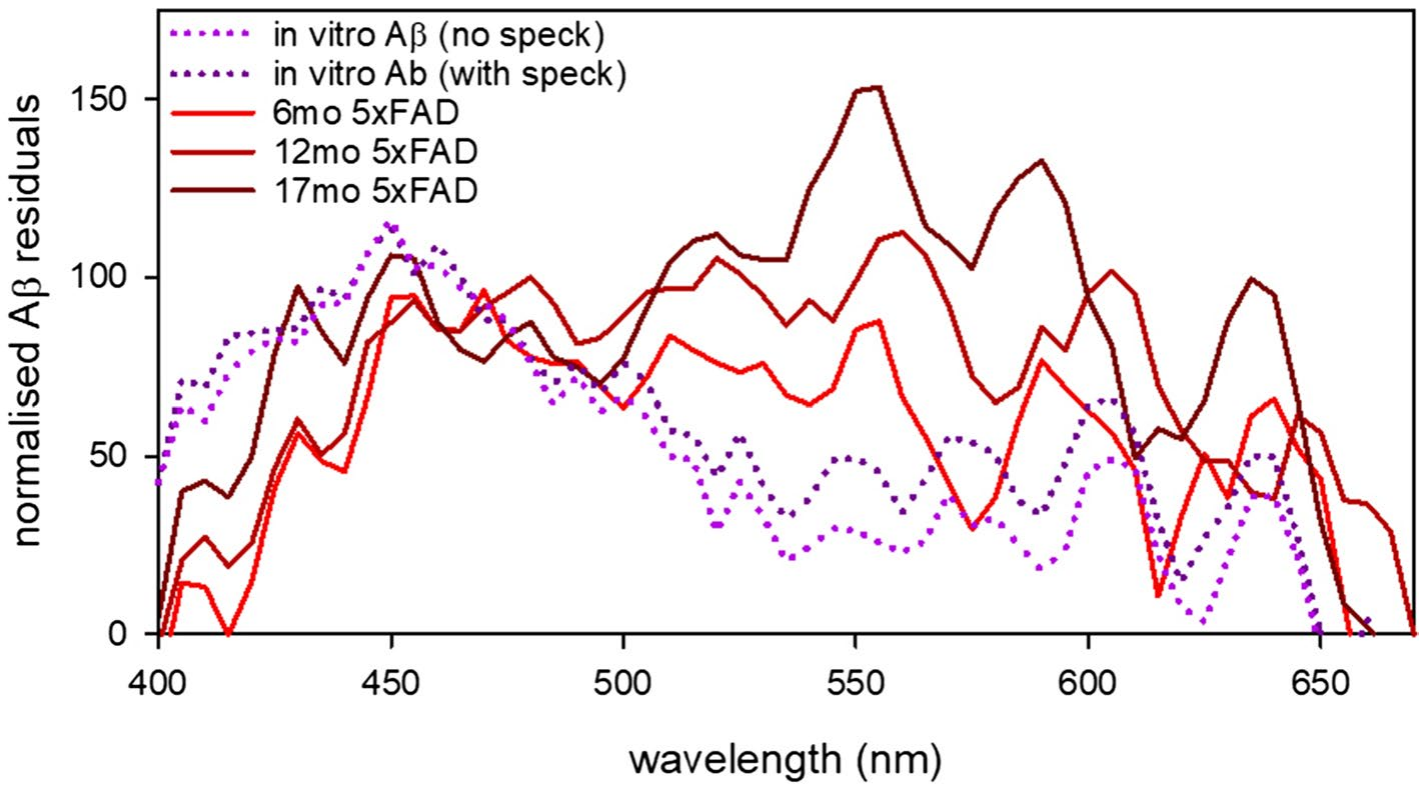
Normalised residual plots showcasing difference between control and amyloid in in vitro and in vivo conditions. Data normalised to a band between 450 and 475 nm to visualise profiles between different preparations.

The main differences in retinal reflectance between 6–12 5xFAD and WT mice appeared around shorter wavelengths (< 500 nm) as well as a second peak and longer wavelengths ~ 550–560 nm. The increased reflectance at short wavelengths is consistent with the in vitro data seen in Fig. 1. The reflectance difference at short wavelengths was less apparent at 17 months of age, and at this age the difference at longer wavelengths (> 550 nm) was more apparent. Difference between 5xFAD mice of different ages are most obvious when the residuals (Aβ – Control) are plotted along the same axis (Fig. 5). In order to more easily visualize the ageing shift towards increased reflectance at longer wavelengths, Fig. 5 shows the residuals normalized to an approximate band around the short wavelength peaks of different ages and preparations (average residual between 450–475 nm). This approach also facilitates comparison of in vivo and in vitro Aβ reflectivity.

## Discussion

### Change in hyperspectral reflectivity at short wavelengths

This study shows increased relative reflectance at shorter wavelengths (< 500 nm) may be a signature of retinal changes associated with different Aβ preparations. This was relatively consistent when we compared hyperspectral imaging of isolated human Aβ42 (in vitro) in a drop of water to in vivo retinal imaging in a transgenic murine model shown to exhibit Aβ in the retina. That the presence of Aβ increases reflectance at shorter wavelengths is consistent with a recent clinical study reporting that participants with mild cognitive impairment and high cortical Aβ load (PET imaging) exhibited increased reflectance at short wavelengths compared to age matched controls^30^. That study modelled optical media, posterior pole pigmentation, and haemoglobin (using Dimension Reduction by Orthogonal Projection for Discrimination, DROP-D) all of which modify retinal reflectance between individuals. The need for such modelling may be less important in rodent eyes given the greater homogeneity within an animal model and the absence of macular pigmentation. It is of note that increased reflectance in 5xFAD mice was less apparent at 17 months of age than at 6 and 12 months (Fig. 4), which may be due to absorbance of shorter wavelengths by the crystalline lens^51^.

More and colleagues reported a decrease in reflectance of short wavelengths ~ 480 to 550 nm, in a human neuroblastoma cell line^27^ and in APP/PS1 AD mice^28^. This is an effect opposite to that observed in the current study and that of Hadoux et al.^30^ The current study and Hadoux et al.^30^ employed perpendicular bright field illumination whereas More and Vince^27^ used a system analogous to a darkfield microscope. This different lighting and imaging geometry might explain why their system detected reduced reflectance at the short wavelengths with increasing Aβ42 aggregation. In contrast, our and Hadoux et al.’s^30^ approach employing a simple ophthalmoscope, resulted in increased reflectance at short wavelengths.

Using a LED based interferometric system with broadband incident light at 0°, Cheng et al.^52^ showed a shift towards increased total reflectance associated with increased soluble Aβ. Reflectance was further increased by the addition of fibril promoting metal compounds (e.g. zinc), whereas reduced reflectance was seen in the presence of polyphenols (e.g. (–)-epigallocatechin-3-gallate), which prevents fibril elongation. In another study, Yu et al.^53^, using a spectrofluorometer with a light source placed directly behind the sample (0° incident beam) showed that particles (gold-coated iron oxide) with diameters of 320 nm in the UV–VIS range when coated with increasing concentrations of Aβ resulted in increasing reflectance.

### Hyperspectral imaging with advancing age

Figures 4 and 5 illustrate how the shape of the HSI profile modifies with age. All profiles show an increase in reflectance at shorter wavelengths. By normalising the residuals to a band between 450 and 475 it can be seen that with advancing age the 5xFAD mice show a relatively greater reflection at longer wavelengths as well. A previous study by our group quantified immunohistochemical staining of Aβ plaques and oligomers in a sub-set of these 5xFAD mice^22^. This study showed that oligomer levels were highest at 6 months of age and then gradually declined at 12 and 14–17 months of age. In contrast Aβ plaque deposition gradually increased with advancing age. This pattern was mirrored in hippocampal and cortical tissues. Age-matched control tissue exhibited lower levels of Aβ staining than tissue from 5xFAD counterparts. These findings support the idea that younger mice exhibit increased reflectance at shorter wavelengths corresponding to oligomer induced light scatter (Figs. 4a, 5). With advancing age, the increased reflectance (Figs. 4b,c, 5) at short wavelengths is less prominent and reflectance at longer wavelengths (> 550 nm) increases, which may be associated with Aβ plaque induced scatter.

This study also examines the spectral profile of human Aβ42 when suspended in PBS. We show that amyloid is consistent with a light scattering effect, resulting in increased light reflectance at shorter wavelengths of the visible light spectrum (Fig. 1). When comparing those ROIs in the in vitro preparation that included visible aggregates and background solution versus those ROIs where only the background solution was visible produced similar profiles. There was a trend for those ROIs which included visible specks to have increased reflectivity at longer wavelengths consistent with the 5xFAD data’s progression with advancing age however this trend was minimal and not significant.

## Limitations

When considering in vivo spectroscopy, it is also possible that other biological factors come into play. The 5xFAD data were normalised to a long wavelength based on previous literature of Aβ-aggregates in cell culture^27^, excised brain and retinal tissue^27^, in-vivo mouse tissue^28^, and our in vitro data (Fig. 1). However, for completeness and to additionally consider other non-Aβ influences on the HSI profiles the data was also normalized to the average reflectance of all the wavelengths (Supplementary Fig. 3). In this manner, variability from differences in overall reflectivity can be removed whilst making no assumptions as to what is causing reflectivity changes or at what wavelengths they occur. This alternate approach (Supplementary Fig. 3) shows a similar shaped profile to Fig. 4, albeit shifted downwards. Regardless of the normalisation approach, the observation that at shorter wavelengths the 5xFAD mice show increased reflectivity; and with advancing age there is an additional peak at ~ 550 nm remains consistent. However, the approach of normalising to the average reflectivity shows a reduction in reflectivity at long wavelengths (> 600 nm) not seen when normalised to 670 nm.

Altered oxygenation causes changes to retinal reflectivity in this wavelength range. 600–750 nm^54^. Although major blood vessels were masked, retinal capillaries spread across the retina making altered tissue oxygenation a possible driver of changes seen at wavelengths above 600 nm. In a subset of animals, partial pressure of oxygen (PO2) was measured and no significant difference were noted at any age between 5xFAD mice and WT littermates (Supplementary Table 3), making this explanation perhaps less likely. Note that pO2 analysis was conducted under general anesthesia analogous to that used during hyperspectral imaging. Nevertheless, to conclusively determine whether altered oxygenation is driving the hyperspectral changes at long wavelengths or whether it is a mathematical manifestation of normalising to the centre of the spectrum as opposed to the end requires further interventional studies such as hyperoxic breathing.

Another important factor to consider with retinal imaging is the effect of age-related cataract formation in older eyes, which can mask light scatter at shorter wavelengths. Although age-matched control animals were used as a comparison it is possible that 5xFAD mice may exhibit greater anterior eye opacities and thus contribute to the difference in the 17-month-old age group compared with younger 5xFAD mice. Additionally, the retinal nerve fibre layer (RNFL) and in particular the microtubules within ganglion cell axons reflect a significant amount of light in the short (415–440 nm) visible wavelength spectrum^55–58^. In humans with AD and animal models of the disease, RNFL thinning has been reported^59–62^. However, RNFL thinning should reduce reflectance between 415 and 440 nm, which is inconsistent with the increased reflectance observed in this study. Nevertheless, to determine the exact contributions of increased short and mid wavelength reflectivity, future interventional studies are required.

## Conclusions

We describe in this study a simple and non-invasive biomarker for the detection of amyloid-related changes in a murine model of AD. This was achieved without the administration of a contrast agent and did not require complex mathematical modelling. Our observations in younger 5xFAD mice suggest that increased reflectance towards shorter wavelengths coincides with when oligomer levels are highest in the retina. With advancing age, the reflectance at longer wavelengths increases when Aβ plaques show greater deposition in the retina. Future studies are required to determine whether this observation is causal in nature, and whether it may be a useful metric for staging Alzheimer’s disease.

## Supplementary information

Supplementary information.

## Data Availability

The authors confirm that we will adhere to the journals data availability policies including making materials, data and associated protocols promptly available to readers without undue qualifications in material transfer agreements. The authors also disclose a subset of the animals used in this manuscript had separate techniques conducted on them (electroretinography, optical coherence tomography) which was published in another manuscript (Lim et al. 2020, Frontier in Neuroscience) as well as their tissue taken and analysed (Habiba et al. 2020, Journal of Alzheimer's Disease).
