# Supplementary material for: Retinal hyperspectral imaging in the 5xFAD mouse model of Alzheimer’s disease
Source: Sci Rep. 2021 Mar 18;11:6387. doi: 10.1038/s41598-021-85554-2 (PMC7973540; doi:10.1038/s41598-021-85554-2)
Supplement: Supplementary file 1 — Supplementary information. [file 41598_2021_85554_MOESM1_ESM.docx]

**Supplementary information: Retinal hyperspectral imaging in the 5xFAD mouse model of Alzheimer’s disease**

*Jeremiah K.H. Lim^1,4^, Qiao-Xin Li^2^, Tim Ryan^3^, Phillip Bedggood^1^, Andrew Metha^1^, Algis J. Vingrys^1^, Bang V. Bui^1^, Christine T.O. Nguyen^1*^*

^1^ Department of Optometry and Vision Sciences, University of Melbourne, Parkville, 3010, Victoria, Australia.

^2^Florey Institute of Neuroscience and Mental Health, Parkville, 3010, Victoria, Australia.

^3^SAXS/WAXS Beamline, ANSTO/Australian Synchrotron, Clayton, 3168, Australia

^4^Optometry and Vision Science, College of Nursing and Health Sciences, Flinders University, Bedford Park, 5042, South Australia, Australia.

*corresponding author: [christine.nguyen@unimelb.edu.au](mailto:christine.nguyen@unimelb.edu.au)


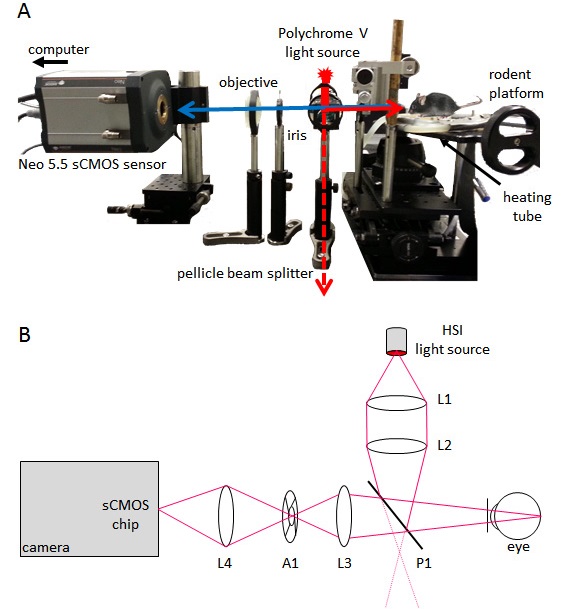


Supplementary Figure 1. Optical bench ophthalmoscope used for hyperspectral imaging. A. shows a photograph of the hyperspectral optic bench setup. Red lines show the pathway of light entering the mouse eye. Blue lines show the light returning from the retina into the sensor chip of the camera. B. shows a schematic of the light path (pink lines). Lenses are marked by Ln. A1 marks the iris aperture used to limit unwanted incident light, P1 marks the pellicle beam-splitter used to divert incident light into the retina. A glass coverslip is placed parallel to the corneal plane and held in place by Genteal gel, which doubles as a coupling medium and to prevent ocular surface dehydration. For in vitro preparations the solution is placed at the same plane and location as the eye in this schematic.

Supplementary Figure 2. Representative Aβ spectra before and after correction for light output and spectral attenuation by semi-silvered pellicle. A. shows the light input spectrum measured using a digital power meter and normalised to 670 nm. B. shows the pellicle transmittance profile as specified by the manufacturer. C. shows a representative raw *in vitro* hyperspectral profile of Aβ42 D. shows a representative raw *in vivo* 5xFAD retinal hyperspectral profile E. shows the corrected *in vitro* profile F. shows the corrected *in vitro* intensity

Supplementary Figure 3*:* Hyperspectral profile of 5xFAD normalised to average of all wavelengths *In vivo* imaging of 5xFAD and WT retinae using hyperspectral imaging (n = 8-15 / group) shows a separation between 5xFAD (red trace) and WT (blue trace) at 6 (*A*), 12 (*B*) and 17 (*C*) months of age. This difference is plotted as residuals (green line). ** significant difference using two-way ANOVA; Grey shaded regions, 95% confidence limits of WT only to aid visualisation.*

**Supplementary Table 1. Statistics for in vitro Aβ versus vehicle**

Two way repeated measures ANOVA where, DF (degrees of freedom), SS (sum of squares), MS (mean square), and post-hoc comparisons between treatments. ***A.*** Aβ with no visible aggregates (no specks) ***B.*** Aβ with visible aggregates (with specks)

| ***A***. Aβ (no specks) | DF | SS | MS | F-value | P-value |
| --- | --- | --- | --- | --- | --- |
| interaction | 54 | 13367 | 247.5 | 7.40 | <0.0001 |
| wavelength | 54 | 393689 | 7291 | 217.6 | <0.0001 |
| Aβ | 1 | 26662 | 26662 | 4.64 | 0.0450 |
| Subject | 18 | 103397 | 5744 | 171.5 | <0.0001 |
| residual | 972 | 32562 | 33.50 |  |  |

| Post-hoc | Mean diff | Discovery | P-value |
| --- | --- | --- | --- |
| 400 | -8.980 | No | 0.1885 |
| 405 | -13.52 | No | 0.0551 |
| 410 | -12.55 | No | 0.1059 |
| 415 | -15.39 | Yes | 0.0491 |
| 420 | -16.65 | Yes | 0.0252 |
| 425 | -17.45 | Yes | 0.0232 |
| 430 | -17.20 | Yes | 0.0201 |
| 435 | -19.42 | Yes | 0.0062 |
| 440 | -19.53 | Yes | 0.0057 |
| 445 | -22.50 | Yes | 0.0029 |
| 450 | -24.46 | Yes | 0.0014 |
| 455 | -21.58 | Yes | 0.0031 |
| 460 | -21.76 | Yes | 0.0021 |
| 465 | -20.39 | Yes | 0.0045 |
| 470. | -19.53 | Yes | 0.0059 |
| 475 | -18.46 | Yes | 0.0042 |
| 480 | -15.95 | Yes | 0.0054 |
| 485 | -13.49 | Yes | 0.0180 |
| 490 | -15.23 | Yes | 0.0078 |
| 495 | -13.06 | Yes | 0.0177 |
| 500 | -14.10 | Yes | 0.0133 |
| 505 | -12.77 | Yes | 0.0392 |
| 510 | -10.49 | No | 0.0670 |
| 515 | -10.25 | No | 0.0527 |
| 520 | -6.021 | No | 0.2452 |
| 525 | -8.977 | No | 0.1162 |
| 530 | -6.920 | No | 0.2062 |
| 535 | -4.287 | No | 0.3800 |
| 540 | -5.149 | No | 0.3548 |
| 545 | -6.225 | No | 0.2691 |
| 550 | -6.069 | No | 0.3115 |
| 555 | -5.389 | No | 0.2959 |
| 560 | -4.842 | No | 0.3495 |
| 565 | -5.503 | No | 0.2706 |
| 570 | -8.252 | No | 0.1146 |
| 575 | -6.365 | No | 0.1693 |
| 580 | -6.880 | No | 0.1988 |
| 585 | -5.278 | No | 0.3225 |
| 590 | -3.766 | No | 0.4522 |
| 595 | -4.949 | No | 0.4014 |
| 600 | -9.682 | No | 0.0746 |
| 605 | -10.28 | Yes | 0.0389 |
| 610 | -9.794 | Yes | 0.0495 |
| 615 | -4.710 | No | 0.2873 |
| 620 | -1.563 | No | 0.7071 |
| 625 | -0.7079 | No | 0.8297 |
| 630 | -4.371 | No | 0.3447 |
| 635 | -8.089 | Yes | 0.0062 |
| 640 | -8.110 | Yes | 0.0349 |
| 645 | -4.530 | No | 0.2428 |
| 650 | 1.135 | No | 0.7894 |
| 655 | 4.806 | No | 0.1752 |
| 660 | 2.050 | No | 0.4736 |
| 665 | 1.891 | No | 0.5326 |

| ***B***. Aβ (with specks) | DF | SS | MS | F-value | P-value |
| --- | --- | --- | --- | --- | --- |
| interaction | 54 | 9956 | 184.4 | 6.93 | <0.0001 |
| wavelength | 54 | 418303 | 7746 | 291.2 | <0.0001 |
| Aβ | 1 | 34567 | 34567 | 6.44 | 0.0206 |
| Subject | 18 | 96624 | 5368 | 201.8 | <0.0001 |
| residual | 972 | 25861 | 26.61 |  |  |

| Post-hoc | Mean diff | Discovery | P-value |
| --- | --- | --- | --- |
| 400 | -8.505 | No | 0.1985 |
| 405 | -14.27 | No | 0.0513 |
| 410 | -13.82 | No | 0.0785 |
| 415 | -16.70 | Yes | 0.0357 |
| 420 | -16.88 | Yes | 0.0220 |
| 425 | -17.04 | Yes | 0.0229 |
| 430 | -17.12 | Yes | 0.0146 |
| 435 | -19.43 | Yes | 0.0032 |
| 440 | -18.79 | Yes | 0.0033 |
| 445 | -21.46 | Yes | 0.0011 |
| 450 | -22.80 | Yes | 0.0002 |
| 455 | -20.13 | Yes | 0.0011 |
| 460 | -21.90 | Yes | 0.0005 |
| 465 | -19.98 | Yes | 0.0018 |
| 470. | -17.61 | Yes | 0.0036 |
| 475 | -17.56 | Yes | 0.0011 |
| 480 | -15.49 | Yes | 0.0020 |
| 485 | -14.00 | Yes | 0.0046 |
| 490 | -15.23 | Yes | 0.0023 |
| 495 | -13.45 | Yes | 0.0053 |
| 500 | -15.22 | Yes | 0.0027 |
| 505 | -14.09 | Yes | 0.0105 |
| 510 | -11.43 | Yes | 0.0351 |
| 515 | -11.15 | Yes | 0.0269 |
| 520 | -8.803 | No | 0.0906 |
| 525 | -11.28 | Yes | 0.0452 |
| 530 | -8.313 | No | 0.1160 |
| 535 | -6.442 | No | 0.1904 |
| 540 | -7.588 | No | 0.1713 |
| 545 | -9.761 | No | 0.0767 |
| 550 | -9.885 | No | 0.0903 |
| 555 | -9.071 | No | 0.1061 |
| 560 | -6.831 | No | 0.2530 |
| 565 | -8.718 | No | 0.1256 |
| 570 | -11.11 | Yes | 0.0307 |
| 575 | -10.74 | Yes | 0.0292 |
| 580 | -10.10 | No | 0.0695 |
| 585 | -7.462 | No | 0.1757 |
| 590 | -6.648 | No | 0.2043 |
| 595 | -9.618 | No | 0.0894 |
| 600 | -12.79 | Yes | 0.0092 |
| 605 | -13.25 | Yes | 0.0038 |
| 610 | -11.32 | Yes | 0.0191 |
| 615 | -6.374 | No | 0.1360 |
| 620 | -2.901 | No | 0.5071 |
| 625 | -5.389 | No | 0.1341 |
| 630 | -7.093 | No | 0.1183 |
| 635 | -10.01 | Yes | 0.0008 |
| 640 | -9.939 | Yes | 0.0120 |
| 645 | -5.441 | No | 0.1778 |
| 650 | -0.09999 | No | 0.9828 |
| 655 | 2.168 | No | 0.5538 |
| 660 | -0.7520 | No | 0.8165 |
| 665 | 2.993 | No | 0.3100 |

**Supplementary Table 2. Statistics for WT versus 5xFAD mice**

Two way repeated measures ANOVA where, DF (degrees of freedom), SS (sum of squares), MS (mean square), and post-hoc comparisons between treatments. ***A.*** 6 months old

***B.*** 12 months old ***C.*** 17 months old

| ***A***. 6mo | DF | SS | MS | F-value | P-value |
| --- | --- | --- | --- | --- | --- |
| interaction | 54 | 378.2 | 7.004 | 3.31 | <0.0001 |
| wavelength | 54 | 500117 | 9261 | 4378 | <0.0001 |
| genotype | 1 | 1203 | 1203 | 4.05 | 0.0602 |
| subject | 17 | 5043 | 296.6 | 140.2 | <0.0001 |
| residual | 918 | 1942 | 2.116 |  |  |

| Post-hoc | Mean diff | Discovery | P-value |
| --- | --- | --- | --- |
| 400 | 0.6200 | No | 0.5879 |
| 405 | -0.5756 | No | 0.6299 |
| 410 | -0.5304 | No | 0.6738 |
| 415 | 0.01143 | No | 0.9930 |
| 420 | -0.5840 | No | 0.6637 |
| 425 | -1.654 | No | 0.2195 |
| 430 | -2.262 | No | 0.1072 |
| 435 | -1.952 | No | 0.1762 |
| 440 | -1.829 | No | 0.2330 |
| 445 | -2.677 | No | 0.0987 |
| 450 | -3.786 | Yes | 0.0240 |
| 455 | -3.809 | Yes | 0.0197 |
| 460 | -3.423 | Yes | 0.0396 |
| 465 | -3.416 | No | 0.0579 |
| 470. | -3.874 | Yes | 0.0365 |
| 475 | -3.304 | No | 0.0544 |
| 480 | -3.116 | Yes | 0.0415 |
| 485 | -3.040 | Yes | 0.0241 |
| 490 | -3.067 | Yes | 0.0341 |
| 495 | -2.778 | No | 0.0745 |
| 500 | -2.545 | No | 0.1294 |
| 505 | -2.888 | No | 0.0600 |
| 510 | -3.359 | Yes | 0.0190 |
| 515 | -3.194 | Yes | 0.0264 |
| 520 | -3.054 | Yes | 0.0475 |
| 525 | -2.945 | No | 0.0608 |
| 530 | -3.045 | Yes | 0.0469 |
| 535 | -2.686 | No | 0.0883 |
| 540 | -2.578 | No | 0.0813 |
| 545 | -2.754 | No | 0.0529 |
| 550 | -3.425 | Yes | 0.0245 |
| 555 | -3.521 | Yes | 0.0205 |
| 560 | -2.659 | No | 0.0770 |
| 565 | -2.222 | No | 0.1362 |
| 570 | -1.697 | No | 0.2163 |
| 575 | -1.183 | No | 0.3860 |
| 580 | -1.539 | No | 0.2097 |
| 585 | -2.405 | Yes | 0.0450 |
| 590 | -3.074 | Yes | 0.0165 |
| 595 | -2.776 | Yes | 0.0364 |
| 600 | -2.514 | No | 0.0532 |
| 605 | -2.275 | Yes | 0.0340 |
| 610 | -1.850 | No | 0.0539 |
| 615 | -0.4260 | No | 0.6719 |
| 620 | -1.323 | No | 0.1058 |
| 625 | -2.031 | Yes | 0.0160 |
| 630 | -1.532 | No | 0.0881 |
| 635 | -2.458 | Yes | 0.0117 |
| 640 | -2.645 | Yes | 0.0067 |
| 645 | -2.092 | Yes | 0.0234 |
| 650 | -1.743 | Yes | 0.0203 |
| 655 | -0.3313 | No | 0.7019 |
| 660 | 0.9357 | No | 0.3324 |
| 665 | 0.7085 | No | 0.1433 |

| ***B***. 12mo | DF | SS | MS | F-value | P-value |
| --- | --- | --- | --- | --- | --- |
| interaction | 54 | 312.6 | 5.789 | 0.65 | 0.9775 |
| wavelength | 54 | 565401 | 10470 | 1170 | <0.0001 |
| genotype | 1 | 1874 | 1874 | 4.76 | 0.0426 |
| subject | 18 | 7080 | 393.3 | 43.96 | <0.0001 |
| residual | 972 | 8696 | 8.947 |  |  |

| Post-hoc | Mean diff | Discovery | P-value |
| --- | --- | --- | --- |
| 400 | 0.1338 | No | 0.9399 |
| 405 | -0.7556 | No | 0.5347 |
| 410 | -1.000 | No | 0.4750 |
| 415 | -0.6922 | No | 0.7091 |
| 420 | -0.9287 | No | 0.5907 |
| 425 | -1.710 | No | 0.1638 |
| 430 | -2.212 | No | 0.1049 |
| 435 | -1.846 | No | 0.2052 |
| 440 | -2.064 | No | 0.2084 |
| 445 | -3.000 | Yes | 0.0268 |
| 450 | -3.202 | Yes | 0.0386 |
| 455 | -3.424 | Yes | 0.0461 |
| 460 | -3.129 | No | 0.0733 |
| 465 | -3.114 | No | 0.1046 |
| 470. | -3.362 | No | 0.0786 |
| 475 | -3.510 | Yes | 0.0353 |
| 480 | -3.670 | Yes | 0.0245 |
| 485 | -3.393 | Yes | 0.0335 |
| 490 | -2.983 | No | 0.1000 |
| 495 | -3.046 | No | 0.1384 |
| 500 | -3.269 | No | 0.0999 |
| 505 | -3.519 | Yes | 0.0347 |
| 510 | -3.552 | No | 0.0555 |
| 515 | -3.553 | No | 0.0763 |
| 520 | -3.864 | No | 0.0753 |
| 525 | -3.705 | No | 0.1039 |
| 530 | -3.477 | No | 0.1229 |
| 535 | -3.167 | No | 0.0928 |
| 540 | -3.431 | Yes | 0.0385 |
| 545 | -3.221 | No | 0.1377 |
| 550 | -3.632 | No | 0.1640 |
| 555 | -4.060 | No | 0.1225 |
| 560 | -4.129 | No | 0.1015 |
| 565 | -3.900 | No | 0.1045 |
| 570 | -3.370 | No | 0.0854 |
| 575 | -2.654 | No | 0.0635 |
| 580 | -2.377 | No | 0.1244 |
| 585 | -2.534 | No | 0.2234 |
| 590 | -3.149 | No | 0.2354 |
| 595 | -2.912 | No | 0.2896 |
| 600 | -3.509 | No | 0.1606 |
| 605 | -3.731 | No | 0.1103 |
| 610 | -3.500 | No | 0.0773 |
| 615 | -2.555 | No | 0.0609 |
| 620 | -2.096 | Yes | 0.0286 |
| 625 | -1.786 | No | 0.1592 |
| 630 | -1.789 | No | 0.3961 |
| 635 | -1.462 | No | 0.5868 |
| 640 | -1.389 | No | 0.6032 |
| 645 | -2.251 | No | 0.3810 |
| 650 | -2.073 | No | 0.3707 |
| 655 | -1.376 | No | 0.5408 |
| 660 | -1.340 | No | 0.5168 |
| 665 | -1.056 | No | 0.4386 |

| ***C.*** 17mo | DF | SS | MS | F-value | P-value |
| --- | --- | --- | --- | --- | --- |
| interaction | 54 | 361.1 | 6.687 | 1.52 | 0.0107 |
| wavelength | 54 | 552413 | 10230 | 2317 | <0.0001 |
| genotype | 1 | 1780 | 1780 | 5.70 | 0.0269 |
| subject | 20 | 6241 | 312.0 | 70.69 | <0.0001 |
| residual | 1080 | 4767 | 4.414 |  |  |

| Post-hoc | Mean diff | Discovery | P-value |
| --- | --- | --- | --- |
| 400 | -0.1377 | No | 0.9373 |
| 405 | -1.214 | No | 0.3969 |
| 410 | -1.298 | No | 0.4093 |
| 415 | -1.162 | No | 0.5586 |
| 420 | -1.504 | No | 0.4681 |
| 425 | -2.386 | No | 0.1787 |
| 430 | -2.950 | No | 0.0621 |
| 435 | -2.573 | No | 0.1190 |
| 440 | -2.297 | No | 0.2216 |
| 445 | -2.857 | No | 0.1299 |
| 450 | -3.214 | No | 0.0645 |
| 455 | -3.185 | Yes | 0.0415 |
| 460 | -2.650 | No | 0.1024 |
| 465 | -2.408 | No | 0.2131 |
| 470. | -2.311 | No | 0.2453 |
| 475 | -2.527 | No | 0.1449 |
| 480 | -2.648 | No | 0.0689 |
| 485 | -2.348 | No | 0.0833 |
| 490 | -2.265 | No | 0.1455 |
| 495 | -2.123 | No | 0.2505 |
| 500 | -2.341 | No | 0.2250 |
| 505 | -2.771 | No | 0.1134 |
| 510 | -3.156 | Yes | 0.0499 |
| 515 | -3.338 | Yes | 0.0316 |
| 520 | -3.393 | Yes | 0.0398 |
| 525 | -3.218 | No | 0.0799 |
| 530 | -3.178 | No | 0.1133 |
| 535 | -3.178 | No | 0.1063 |
| 540 | -3.780 | Yes | 0.0386 |
| 545 | -4.122 | Yes | 0.0156 |
| 550 | -4.610 | Yes | 0.0119 |
| 555 | -4.645 | Yes | 0.0113 |
| 560 | -4.026 | Yes | 0.0226 |
| 565 | -3.458 | No | 0.0594 |
| 570 | -3.305 | No | 0.0771 |
| 575 | -3.103 | No | 0.0614 |
| 580 | -3.594 | Yes | 0.0164 |
| 585 | -3.871 | Yes | 0.0111 |
| 590 | -4.021 | Yes | 0.0136 |
| 595 | -3.664 | Yes | 0.0312 |
| 600 | -2.846 | No | 0.0833 |
| 605 | -2.454 | No | 0.1224 |
| 610 | -1.494 | No | 0.2940 |
| 615 | -1.744 | No | 0.1256 |
| 620 | -1.657 | No | 0.0630 |
| 625 | -1.982 | Yes | 0.0207 |
| 630 | -2.657 | Yes | 0.0291 |
| 635 | -3.013 | No | 0.0740 |
| 640 | -2.878 | No | 0.1452 |
| 645 | -1.992 | No | 0.3310 |
| 650 | -0.9483 | No | 0.6310 |
| 655 | -0.2518 | No | 0.8891 |
| 660 | -0.05142 | No | 0.9710 |
| 665 | 0.1357 | No | 0.8589c |

**Supplementary Table 3. Partial pressure of oxygen and pH in WT and 5xFAD mice.**

The pO2 (oxygen partial pressure) and pH did not differ significantly with genotype at 6, 12 nor 17 months of age (WT n = 6 -9, 5xFAD n = 5-7)

| **Age (m)** | **arterial blood-gas analysis** | **WT** | **5xFAD** | **p-value** |
| --- | --- | --- | --- | --- |
| 6 | pO2 (mmHg) | 82.51±4.9 | 72.34±6.7 | 0.228 |
|  | pH | 7.15±0.01 | 7.13±0.04 | 0.551 |
| 12 | pO2 (mmHg) | 95.53 + 8.4 | 83.06 + 5.9 | 0.276 |
|  | pH | 7.12 + 0.02 | 7.15 + 0.03 | 0.46 |
| 17 | pO2 (mmHg) | 87.81 + 1.9 | 79.23 +3.89 | 0.164 |
|  | pH | 7.14 + 0.01 | 7.12 + 0.02 | 0.424 |
